# Sesame Meal: The Ideal Alternative to Soybean Meal for Fattening Beef Cattle—Reducing Nitrogen Excretion and Bolstering Antioxidant Defenses

**DOI:** 10.3390/antiox14111336

**Published:** 2025-11-06

**Authors:** Shengnan Min, Yingqi Li, Changxiao Shi, Huili Wang, Hongliang Zhang, Shuo Zhang, Yawen Luo, Yan Lu, Yang He, Binghai Cao, Huawei Su

**Affiliations:** State Key Laboratory of Animal Nutrition and Feeding, College of Animal Science and Technology, China Agricultural University, Beijing 100193, China; b20253040538@cau.edu.cn (S.M.); b20243040446@cau.edu.cn (Y.L.); scx1107@cau.edu.cn (C.S.); s20223040747@cau.edu.cn (H.W.); 17830887172@cau.edu.cn (H.Z.); zhangshuocast@cau.edu.cn (S.Z.); s20243040849@cau.edu.cn (Y.L.); sy20233040924@cau.edu.cn (Y.L.); he.yang@cau.edu.cn (Y.H.); caobh@cau.edu.cn (B.C.)

**Keywords:** sesame meal, nitrogen utilization, antioxidant activity, rumen microbiota, sustainable ruminant nutrition

## Abstract

Sesame meal possesses high crude protein content (40–50%), abundant methionine, and natural antioxidant components such as lignan compounds, making it a high-quality feed alternative to soybean meal in ruminant production. This study systematically evaluated the effects of completely replacing soybean meal with sesame meal in the diet on growth performance, serum biochemistry, antioxidant activity, rumen fermentation parameters, and microbial composition in finishing beef cattle. The trial employed a completely randomised design, selecting 18 Angus bulls with similar initial body weights (566.7 ± 38.1 kg). Animals were randomly assigned to the SBM group (*n* = 9) and SSM group (*n* = 9), with a 7-day pre-trial period followed by a 96-day main trial period. Results indicate that replacing soybean meal with sesame meal significantly enhances the antioxidant capacity of fattening beef cattle. Catalase (CAT) activity markedly increased (*p* < 0.05), while glutathione peroxidase activity showed an upward trend (0.05 < *p* < 0.1). This improvement was accompanied by a substantial shift in rumen microbial composition, highlighted by a marked enrichment of beneficial bacteria including p_Verrucomicrobiota, p_Spirochaetota, *g_CAG_352*, *norank_f_Lachnospiraceae*, and *g_Rikenellaceae_RC9_gut_group*, which collectively contributed to greater microbial complexity and stability. Regarding nitrogen metabolism, urinary nitrogen and serum urea nitrogen levels were significantly reduced in the sesame meal group (*p* < 0.05), indicating improved nitrogen utilization efficiency. Overall, completely replacing soybean meal with sesame meal in the diet of finishing beef cattle did not adversely affect growth and slaughter performance. It simultaneously significantly enhanced antioxidant capacity, reduced urinary nitrogen excretion, and lowered feed costs. These findings underscore the potential of sesame meal as a sustainable, nutritionally advantageous alternative for optimising beef cattle diets.

## 1. Introduction

In modern intensive livestock production, feed costs account for approximately 55% to 70% of total breeding expenses [1]. The quality and price of protein feed directly influence the economic viability of livestock operations. Soybean meal has traditionally been the primary plant protein source in ruminant diets owing to its high crude protein (44–49%) and balanced amino acid content, particularly its lysine content (3.09–3.35%) [2,3,4]. These nutritional attributes make soybean meal a critical component of livestock feed [5]. However, rising prices driven by the international trade fluctuations and climate change have highlighted the need for sustainable and cost-effective alternatives to soybean meal [6].

Sesame meal, a byproduct of sesame oil extraction, has emerged as a promising alternative. Sesame is one of the oldest oilseed crops, cultivated for over 5000 years, and China is a leading global producer, contributing 7.2% of the world’s output despite occupying only 2.0% of the cultivation area [7,8]. The sesame meal is rich in crude protein (40–60%) and contains higher methionine levels (6.97%) compared to soybean meal (0.63–0.76%) [3,9,10]. Methionine, the first limiting amino acid in ruminants [11], and plays a crucial role in protein synthesis, antioxidant defense, and methyl group donation [12,13,14]. Its deficiency can impair protein synthesis, leading to excess amino acids being metabolized in the liver and excreted as urea, resulting in nitrogen loss [15,16]. Additionally, sesame meal is rich in bioactive compounds such as lignans (0.3–1.5%) and polyunsaturated fatty acids (PUFAs) [17,18,19]. Lignans are a class of secondary metabolites widely distributed in plants, formed by the polymerization of two molecules of phenylpropanoid derivatives. They possess various physiological activities, such as antioxidant activity [20].According to their solubility characteristics, lignans in sesame can be classified into liposoluble lignans and water-soluble lignans. The former mainly include sesamin, sesamolin, sesaminol, sesamolinol, and sesamolinol, etc. The latter are mainly glycosides formed by the condensation of 1 to 3 glucose moieties with the aglycones of sesaminol, sesamolinol or sesamolin [21,22,23]. During the extraction of sesame oil, liposoluble lignans dissolve and water-soluble lignans remain in the sesame meal [24].Studies have shown that sesame meal supplementation can reduce oxidative stress in ruminants, as evidenced by lower plasma protein carbonyl levels in goats fed sesame meal [25].

Currently, some progress has been made in the application of sesame meal in the diets of pigs, chickens, and sheep. Research shows that sesame meal is rich in standardized ileal digestible amino acids, especially methionine, making it a high-quality alternative to soybean meal in fattening pig diets [26]. Eleazar Pérez-Trejo et al. revealed that substituting soybean meal with 50% and 100% sesame meal in the diets of fattening lambs resulted in increased total yield, with no negative effects on production traits or carcass quality [27]. Adding 12% sesame meal to fattening lamb diets increased total antioxidant capacity and reduced malondialdehyde in muscle. This not only reduced feed costs but also improved animal health [28]. Additionally, Yamauchi et al. reported that the inclusion of 10% sesame meal in the diet enhanced the growth performance of laying hens, while 20% sesame meal also showed no negative effects on these hens [29]. However, studies on the application of sesame meal in beef cattle, particularly during the late fattening stage, remain limited.

Despite its nutritional benefits, the mechanisms by which sesame meal influences ruminal fermentation, microbial community structure, and antioxidant function in ruminants remain poorly understood. This knowledge gap limits its scientific application in ruminant feed formulations. Therefore, this study was designed to comprehensively assess the implications of a full replacement of soybean meal with sesame meal in the diets of finishing beef cattle. Key metrics examined included growth performance, nutrient digestibility, serum antioxidant status, rumen fermentation parameters, and microbial community composition.

## 2. Materials and Methods

### 2.1. Animals, Diets, and Experimental Design

This study was approved by the Animal Care Committee of China Agricultural University (approval number: AW30604202-1-1) and conducted at Ben wang Ranch (Yinchuan, China) from November 2023 to February 2024.

A cohort of 18 Angus bulls (18 months old; initial body weight 566.7 ± 38.1 kg) was included in the trial. The cattle were randomly allocated into two dietary treatments (*n* = 9): a control group receiving a conventional soybean meal-based ration and a treatment group whose diet contained sesame meal as a complete substitute for soybean meal. The sesame meal was supplied by Ba duan International Cultural Exchange Company, Yinchuan, China.

The total mixed ration (TMR) was designed in accordance with NASEM (2016) [30] guidelines to satisfy the nutritional needs for growth. The study included a 7−day adaptation phase and a 96-day formal trial period. To ensure that the daily rations reached the true isocaloric and isonitrogenous levels, the following steps were taken: Firstly, the daily rations were formulated based on the nutritional requirements of beef cattle, and the nutritional and energy composition of each component was calculated. Then, before mixing the feed, the dry matter (DM), crude protein (CP), neutral detergent fiber (NDF), acid detergent fiber (ADF), and crude ash (Ash) of each feed ingredient were determined. Finally, the daily ration formula was adjusted based on the analyzed nutritional components to achieve the target levels of metabolizable energy (ME) and CP, ensuring that the formulas of each experimental group were isocaloric and isonitrogenous. Table 1, presented in the text, shows the measured values of the collected TMR, with ME being the calculated value. Each cattle was housed separately in open-sided pens with ad libitum access to water, and received feed twice per day at 07:30 and 16:00. All pens were sanitized and cleaned prior to the commencement of the experiment.

**Figure 1:**
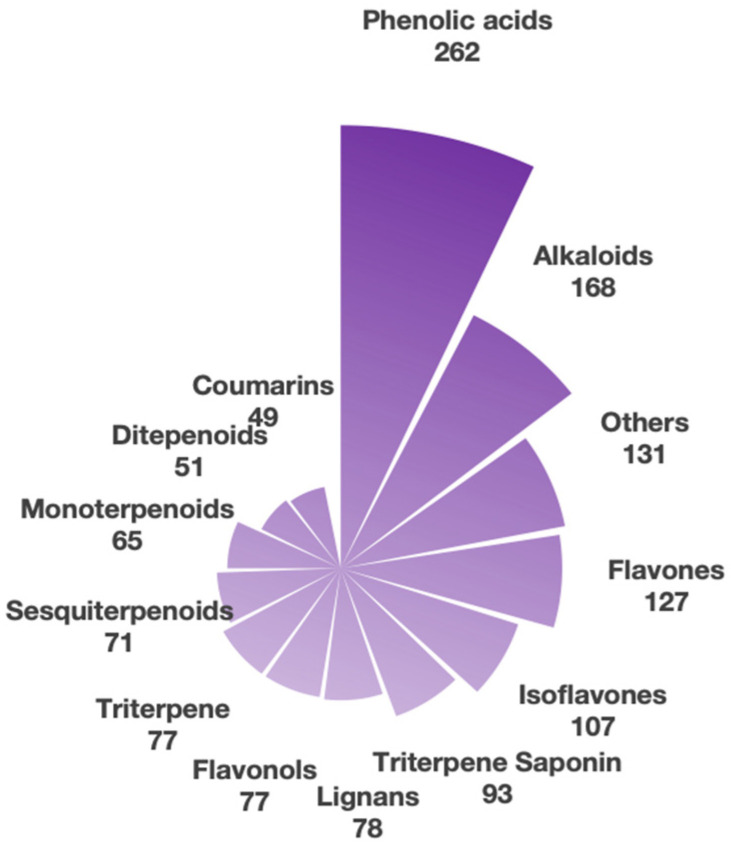
Classification and relative abundance of secondary metabolites in soybean meal and sesame meal.

The amino acid profiles of soybean meal and sesame meal are provided in Table 2. Classification and relative abundance of secondary metabolites in soybean meal and sesame meal are shown in Figure 1. Comparison of Peak Areas for Key Lignin Compounds in Sesame Meal and Soybean Meal in Table 3.

The comparative analysis, based on non-targeted LC-MS metabolomics, highlights the top 13 metabolite classes. Among the 13 major classes detected, lignans emerged as a characteristic and highly abundant bioactive component, with 78 identified compounds—a quantity comparable to that of flavonols and triterpenes (each with 77 compounds) and exceeded only by phenolic acids, alkaloids, and flavonoids.

### 2.2. Sample Collection and Analysis

#### 2.2.1. Growth Performance and Nutrient Digestibility

Initial body weight (IBW) and final body weight (FBW) were measured after fasting, and average daily gain (ADG) was calculated as the difference between FBW and IBW divided by the number of experimental days. Dry matter intake (DMI) was recorded daily, and feed conversion ratio (FCR) was calculated as DMI divided by ADG. Feed samples were collected weekly using the quartering method and pooled monthly for analysis. Fecal samples were collected from the rectum of the bulls using sterile poly-ethylene gloves, over a period of three days (97 d, 98 d, 99 d) and combined into a total of 150 g. This was then mixed with a quarter of 10% tartaric acid to facilitate nitrogen fixation. Feed and fecal samples were dried at 65 °C, ground through a 2 mm sieve, and analyzed for DM, Crude Protein (CP), Ether Extract (EE), Neutral Detergent Fibre (NDF), Acid Detergent Fibre (ADF) and Amino Acids using standard methods [31,32,33]. Apparent nutrient digestibility was determined using acid-insoluble ash (AIA) as an internal marker [34].

#### 2.2.2. Nitrogen Metabolism

Urine samples were collected on day 97 of the formal experimental period, acidified with 0.5 mol/L sulfuric acid to prevent nitrogen volatilization, and stored at −20 °C. Urinary nitrogen content was measured according to AOAC guidelines [31], and creatinine levels were determined using a commercial assay kit (Jiangsu Enzyme Immuno Co., Ltd., Yancheng, China). Total urinary nitrogen excretion was calculated based on creatinine concentration and body weight [35]. The theoretical daily creatinine excretion (g/day) = 0.0345 × SBW^0.9491^, Total urine volume = theoretical creatinine excretion (mmol)/creatinine concentration in urine (mmol/L)

#### 2.2.3. Serum Biochemistry and Antioxidant Activity

Blood samples were collected on day 100 and centrifuged at 3000× *g* for 10 min to obtain serum. Serum biochemical parameters, including glucose (GLU), total protein (TP), albumin (ALB), globulin (GLB), urea nitrogen (UREA), total cholesterol (CHO), creatinine (CREA), triglycerides (TG), non-esterified fatty acids (NEFA), β-hydroxybutyric acid (BHBA), high-density lipoprotein cholesterol (HDL−C), low-density lipoprotein cholesterol (LDL−C), alanine aminotransferase (ALT), aspartate aminotransferase (AST), and alkaline phosphatase (ALP), were analyzed using a Hitachi 7020 biochemistry analyzer (Hitachi Co., Tokyo, Japan). Antioxidant indicators, including catalase (CAT), superoxide dismutase (SOD), total antioxidant capacity (T−AOC), glutathione peroxidase (GSH−Px), malondialdehyde (MDA), and reactive oxygen species (ROS), were measured using commercial kits (Nanjing Jian Cheng Biological Co., Ltd., Nanjing, China and Jiangsu Enzyme Immuno Co., Ltd., Jiangsu, China). The oxidative stress index (OSI) was calculated as ROS/T-AOC.

#### 2.2.4. Rumen Fermentation Parameters

Rumen fluid was collected 3 h after morning feeding on day 100 using a rumen catheter. The pH was immediately measured using a portable pH meter (Testo AG, Schwarzwald, Germany). Samples were filtered through four layers of gauze, divided into aliquots, and stored at −80 °C for volatile fatty acid (VFA) analysis using gas chromatography (GC-2014, Shimadzu Corporation, Kyoto, Japan).

#### 2.2.5. Microbial Community Analysis

Total genomic DNA was extracted from rumen fluid using the E.Z.N.A.^®^ Soil DNA Kit (Omega Biotek, Norcross, GA, USA). The V3-V4 region of the 16S rRNA gene was amplified using barcoded primers 338F (5′-ACTCCTACGGGAGGCAGCAG-3′) and 806R (5′-GGACTACHVGGGTWTCTAAT-3′) [36]. Sequencing was performed on the Illumina PE300/PE250 platform (Shanghai Meiji Biomedical Technology Co., Ltd., Shanghai, China). The sequencing dataset used in this study was obtained from the National Center for Biotechnology Information (NCBI) under the accession code PRJNA1348615. Raw data were analyzed using mothur software to calculate alpha diversity indices (Chao1 and Shannon) and perform principal coordinate analysis (PCoA) based on Bray–Curtis distance [37]. Linear discriminant analysis (LDA) was conducted to identify differentially abundant taxa [38].

#### 2.2.6. Secondary Metabolite Analysis

Grind the freeze-dried samples into powder, add the extraction solution, vortex, centrifuge to obtain the supernatant, filter, and proceed with UPLC-MS/MS analysis. The data acquisition instrumentation primarily comprises ultra-performance liquid chromatography (UPLC) (ExionLC™ AD, https://sciex.com.cn/) and tandem mass spectrometry (MS/MS). Metabolites were identified based on the self-built Metware Database (MWDB) using secondary spectral information for qualitative analysis [39,40].

#### 2.2.7. Slaughter Performance and Meat Sample Collection

Test cattle were fasted for 24 h and deprived of water for 8 h prior to slaughter. Following the established slaughter sequence, the following key indicators were recorded: slaughter order, ante-mortem live weight, kidney and perirenal fat weight, net meat weight, meat color, and pH value. Concurrently, 12–13 kg of 2 kg of the longest muscle from the 12th–13th intercostal lumbar region for subsequent CP and EE chemical composition analysis. Following NY/T 2793−2015 standards, pH values were determined immediately after excision of eye muscle samples using a portable pH meter (Testo 205, Testo AG, Schwarzwald, Germany). After removing external fat and connective tissue, the sample underwent freeze-drying (FD−1−50, Biocool, Beijing, China, −50 °C, 6 days) to determine dry matter content, with the mass ratio before and after freeze-drying calculated. Moisture, crude protein (CP), and crude fat (EE) content were determined according to standard methods.

### 2.3. Statistical Analysis

All data were analyzed using SPSS 27.0 (IBM Corp., Armonk, NY, USA). Independent samples *t*-test was used to compare differences between the SBM and SSM groups. The results are expressed as Mean ± SEM, statistical significance was set at *p* < 0.05, and trends were noted when 0.05 < *p* ≤ 0.10. Multivariate analyses, including Mantel test and redundancy analysis (RDA), were performed in R (version 3.6.3) to assess microbial−physiological correlations.

## 3. Results

### 3.1. Growth Performance

The growth performance of Angus bulls is presented in Table 4. No significant differences were observed between the two groups in terms of IBW, FBW, ADG, DMI, or FCR (*p* > 0.05). These results indicate that the complete replacement of soybean meal with sesame meal did not adversely affect the growth performance of Angus bulls.

### 3.2. Nutrition Digestibility

The apparent digestibility of nutrients is shown in Table 5. No significant differences were detected between the two groups for DM, CP, EE, NDF, or ADF (*p* > 0.05). However, the SSM group exhibited numerically higher digestibility values for DM, EE, NDF, and ADF, suggesting a potential trend toward improved fiber utilization.

### 3.3. Nitrogen Metabolism

Nitrogen metabolism data are presented in Table 6. The SSM group exhibited significantly lower urinary nitrogen excretion compared to the SBM group (*p* = 0.020), while no significant differences were observed in nitrogen intake, fecal nitrogen excretion, nitrogen retention, or nitrogen utilization efficiency (*p* > 0.05). These results indicate that sesame meal supplementation improved nitrogen utilization efficiency, reducing nitrogen loss through urine.

### 3.4. Serum Biochemical Indicators

Serum biochemical parameters are shown in Table 7. The SSM group had significantly lower serum UREA levels compared to the SBM group (*p* = 0.016). Additionally, ALT and ALB levels showed an increasing trend in the SSM group (*p* < 0.10), while other parameters did not differ significantly between the groups (*p* > 0.05).

### 3.5. Serum Antioxidant Capacity

Antioxidant activity data are presented in Table 8. The SSM group exhibited significantly higher CAT activity compared to the SBM group (*p* = 0.043). The GSH−Px activity also showed an increasing trend in the SSM group (*p* = 0.076). No significant differences were observed in the other indices (*p* > 0.05).

### 3.6. Rumen Fermentation Parameters

Rumen fermentation parameters are summarized in Table 9. No significant differences were observed between the two groups in terms of ruminal pH and VFA (*p* > 0.05). These findings suggest that sesame meal substitution did not disrupt rumen fermentation dynamics.

### 3.7. Rumen Microbial Community

**Figure 2:**
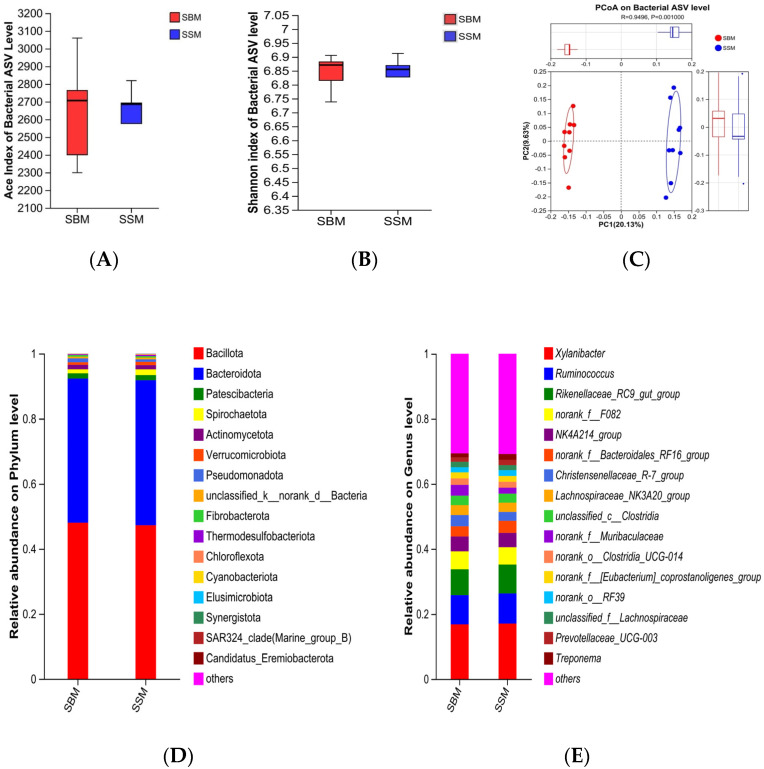
Effects of complete substitution of soybean meal with sesame meal on rumen microbial diversity and composition in Angus bulls. Comparison of bacterial community richness (Ace index) (**A**) and diversity (Shannon index) (**B**) between the SBM and SSM groups. Blue indicates that the group is rich in soybean meal and light red indicates that the group is rich in sesame meal. Comparison of microbial community composition at the phylum (**D**) and genus (**E**) levels for bacteria. Beta diversity PCoA comparison of bacteria (**C**). Box plots show the mean values of richness and diversity, with the box representing the interquartile range, a line in the middle, and two vertical lines at each end representing the maximum and minimum values; Microbial composition across different groups, with each bar indicating the average relative abundance of bacterial taxa within a group. (**D**) Phylum−level taxon allocation; (**E**) Genus−level allocation. Bacterial community composition at the phylum and genus levels is presented, showing the top 16 most abundant taxa; all remaining taxa were grouped into ‘Others’. SBM and SSM represent the soybean meal group and the sesame meal group, respectively.

The rumen microbial community was analyzed using 16S rRNA sequencing (Figure 1). Alpha diversity indices (Ace and Shannon) did not differ significantly between the SSM and SBM groups (Figure 2A,B; Appendix A.2). However, PCoA based on Bray–Curtis distance revealed a significant difference in the microbial community structure between the two groups (*p* < 0.05) (Figure 2C).

**Figure 3:**
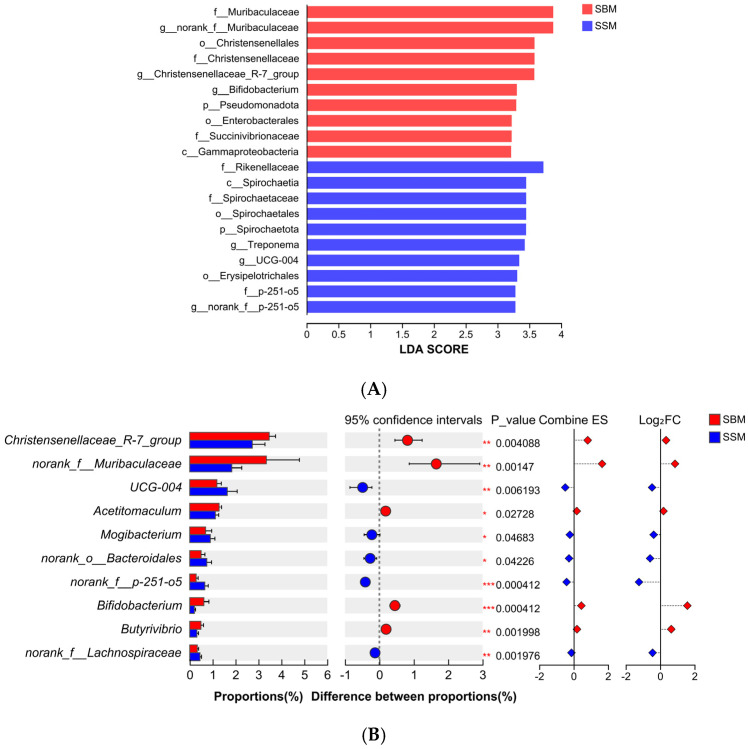
Shows that there are differences in the LDA scores of rumen bacteria between the two groups of diets (LDA > 3). (**A**) presents the LDA score results of the two groups of bacteria, where blue indicates that the group is rich in soybean meal and light red indicates that the group is rich in sesame meal; in (**B**), red indicates that the group is rich in soybean meal and blue indicates that the group is rich in sesame meal. SBM and SSM, respectively, represent the soybean meal group and the sesame meal group. *; *p* < 0.05; **; *p* < 0.01, ***; *p* < 0.001.

At the phylum level, the relative abundance of Spirochaetota was significantly higher in the SSM group (*p* < 0.05) (Figure 2D; Appendix A.3). At the genus level, the SSM group showed significantly higher abundances of *g_UCG-004* and *norank_f_Lachnospiraceae* (*p* < 0.05), *g_Rikenellaceae_RC9_gut_group* was higher than SBM, but the difference was not significant (*p* > 0.05) (Figure 2E; Appendix A.4). Linear discriminant analysis (LDA) further confirmed these differences (Figure 3A).

**Figure 4:**
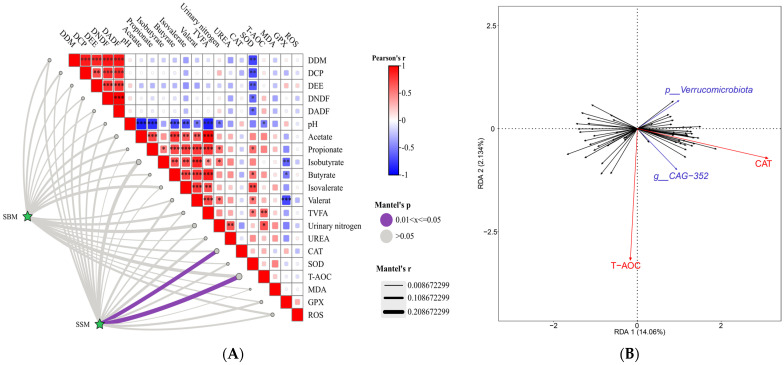
(**A**) presents a heatmap illustrating the correlations between dietary treatments and various indicators in experimental cattle, alongside Mantel test results. This heatmap displays Pearson correlation coefficients between the dietary treatment group (SSM) and each indicator, with red indicating positive correlations and blue denoting negative correlations. Purple lines (*p* < 0.05) mark significant correlations identified by the Mantel test between the dietary treatment matrix and the complete physiological indicator matrix. This figure displays the Mantel r statistic, reflecting the overall strength of association between dietary treatments and the multivariate spectrum of physiological indicators, Mantel test, * *p* < 0.05; ** *p* < 0.01, *** *p* < 0.001. (**B**) RDA plot reveals the separation of rumen microbial communities between the SBM group and the SSM group. Each point represents a sample, with vector lines indicating the direction and strength of correlations between microbial genera and environmental factors. Mantel and RDA analyses were conducted using microbial communities that showed differential abundance (LDA score > 2.5, *p* < 0.05) from LEfSe analysis.

Mantel analysis revealed a significant correlation between rumen microbial community structure and the host’s overall physiological parameter profile (Mantel’s *p* < 0.05). As depicted in Figure 4A, the microbial community matrix based on Bray–Curtis distances exhibited a significant positive correlation with host antioxidant parameters, particularly with CAT activity and T−AOC. RDA results indicate that both the door−level microbial cluster p_Verrucomicrobiota and the genus-level cluster *g_CAG_352* exhibit a positive correlation with CAT activity (Figure 4B).

### 3.8. Slaughter Performance and Meat Quality

The slaughter performance and meat quality of cattle are presented in Table 10. No significant differences were observed between the two groups in any measured parameters (*p* > 0.05). These results indicate that the substitution of soybean meal with sesame meal had no adverse effects on slaughter performance or meat quality.

## 4. Discussion

The complete replacement of soybean meal with sesame meal enhanced body composition in fattening Angus bulls while maintaining growth and slaughter performance. These findings confirm the value of sesame meal as a sustainable alternative protein source in ruminant nutrition [4,41,42,43]. Below, we discuss the implications of our results in the context of nitrogen metabolism, antioxidant capacity, and rumen microbial community dynamics.

### 4.1. Nitrogen Metabolism and Serum Biochemistry

The most notable findings of this study were the significant reduction in urinary nitrogen excretion and serum urea nitrogen in the SSM relative to the SBM. The observed enhancement in nitrogen metabolism was linked to the greater methionine content of sesame meal. Methionine serves as the first limiting amino acid by governing efficient protein synthesis and nitrogen utilization. While the higher methionine content of sesame meal may have contributed to improved post-absorptive nitrogen utilization, we acknowledge that dietary methionine could be partially utilized by ruminal microorganisms unless protected. Therefore, the observed enhancement in nitrogen metabolism is likely attributable to a combination of factors beyond a single amino acid. The protein characteristics of sesame meal, such as its ruminal degradation rate, may have promoted better synchronization of energy and nitrogen release in the rumen, thereby increasing microbial protein synthesis and reducing ammonia production. This improved microbial nitrogen capture would subsequently decrease the flux of urea precursors to the liver. Concurrently, the balanced amino acid profile provided by sesame meal likely further optimized nitrogen retention and reduced hepatic deamination of excess amino acids, consistent with previous reports showing that rumen-protected methionine supplementation lowers nitrogen excretion in dairy cows [44,45,46]. Beyond enhancing feed efficiency, this approach offers significant environmental benefits by reducing urinary nitrogen—a key precursor of greenhouse gas emissions [47]. Similarly, it may also be responsible for the tendency of the SSM group to have elevated serum ALB levels, which maintains the osmotic pressure and acid-base balance of the organism [48], and whose elevation promotes the synthesis and accumulation of proteins, thereby improving the nutritional status of beef cattle [49,50].

### 4.2. Antioxidant Capacity

During the late fattening phase of beef cattle, the use of high-concentrate diets often induces oxidative stress, which commonly requires dietary supplementation with exogenous antioxidants to stimulate the production of endogenous antioxidants like SOD and CAT [51]. In the present study, a marked increase in serum CAT activity was observed in the SSM relative to the SBM, suggesting a boost in antioxidant capacity. This improvement may be attributed to the abundant lignans identified in sesame meal, particularly sesamol and other phenolic compounds. These bioactive compounds, known for their potent antioxidant properties [20,52], may alleviate oxidative stress by scavenging ROS and enhancing endogenous antioxidant defence systems. Furthermore, the elevated methionine content in sesame meal may further bolster antioxidant capacity by promoting the synthesis of glutathione, a key intracellular antioxidant [53]. Relevant studies indicate that methionine supplementation significantly elevates glutathione peroxidase activity in deer serum [54], consistent with the increased glutathione peroxidase activity observed in the SSM group in this study. The synergistic interaction between the lignans inherent in sesame meal and methionine provides a compelling explanation for the enhanced antioxidant status observed in the SSM group. These findings suggest that sesame meal contributes not only as a protein source but also, through its dual role as a methyl donor and natural antioxidant provider, to the overall health and stress resilience of ruminants.

### 4.3. Rumen Microbial Community and Fiber Degradation

Feed digestion and utilization in Ruminantia largely depend on the microbial community within the rumen, which converts potentially digestible feed into metabolizable nutrients for Ruminantia [55]. In this study, Bacteroidota and Bacillota dominated the rumen microbial community as the two most abundant phyla. The relative abundance of these two phyla was significant in both experimental groups, with their combined abundance exceeding 90%. Analysis also revealed a distinct microbial profile between the SBM and SSM groups, marked by differential abundance of fiber-degrading microorganisms such as Spirochaetota, *g_UCG-004*, and *g_Rikenellaceae_RC9_gut_group*. These microbes are known for their roles in degrading complex plant fibers, including cellulose and hemicellulose [56,57,58]. The increased abundance of these taxa in the SSM group may explain the numerically higher fiber digestibility observed in this study, despite non-significant differences. The higher rumen pH in the SSM group likely created a more favorable environment for fiber-degrading microbes, enhancing their activity and contributing to improved feed utilization [59,60]. Furthermore, *g_Rikenellaceae_RC9_gut_group* plays a central role in ruminal fermentation patterns and the development of the ruminal epithelium [61]. G. Conte et al. demonstrated that the *g_Rikenellaceae_RC9_gut_group* was associated with enhanced growth performance in the host animal [62]. Dachio et al. also found that in animals with already optimal growth traits, the *g_Rikenellaceae_RC9_gut_group* exhibited the highest abundance [63]. These findings are consistent with the results of this study and may be a significant factor contributing to the improved growth performance of the experimental bulls in the SSM group. The enrichment of *Lachnospiraceae* in the SSM group is particularly noteworthy, as this family is associated with butyrate production and immune modulation [64,65]. Butyrate contributes significantly to rumen health and function. The presence of *Lachnospiraceae* may also explain the comparable butyrate levels observed in both groups despite the differences in dietary composition. These findings underscore the promise of sesame meal to modulate the rumen microbiome in ways that enhance fiber degradation and overall rumen function.

In this study, correlation analysis revealed that the rumen microbial community structure of beef cattle fed sesame meal (SSM) exhibited a positive correlation with host antioxidant parameters, particularly with CAT activity and T-AOC. Notably, the rumen bacterium *g_CAG-352* (belonging to the Oscillospiraceae family), which was enriched in the SSM group, showed a positive correlation with serum CAT activity, suggesting this bacterium may participate in regulating the host’s antioxidant physiological processes. Although Oscillospiraceae bacteria have been reported [66] to possess metabolic potential for producing short-chain fatty acids (such as butyrate), this study did not detect significant changes in ruminal butyrate concentrations within the SSM group. This finding indicates that enrichment of a single microbial group does not necessarily correlate linearly with end-product metabolite concentrations, potentially influenced by multiple factors, including substrate availability, microbial interactions, or overall host metabolic regulation. Additionally, this study observed an upward trend in bovine serum albumin (ALB) levels within the SSM group, consistent with prior reports of a positive correlation between *g_CAG-352* and ALB [61]. Given that ALB is recognised as an endogenous antioxidant protein, this finding suggests that *g_CAG-352* enrichment may enhance antioxidant defence by directing nitrogen metabolism towards ALB synthesis and synergising with antioxidant enzyme systems (e.g., catalase) [67,68]. While these correlations are consistent with the improved antioxidant status observed in the SSM group, they do not establish causality, and the underlying mechanisms require further validation.

Concurrently, Verrucomicrobiota enrichment was observed in the SSM group. RDA further indicated a positive correlation between this phylum and serum CAT content. Existing research predominantly attributes [69] Verrucomicrobiota to complex polysaccharide degradation, particularly excelling in utilising sulphated methylpentoses (e.g., fucose and rhamnose), a capability validated in marine ecosystems [70]. Within ruminant rumens, Verrucomicrobia may adaptively utilise plant-derived polysaccharides, promoting propionate production via the pentose phosphate pathway. This enhances energy metabolism and stimulates rumen epithelial absorption of propionate. Subsequent hepatic metabolism of propionate may supply energy and precursor substances for synthesising antioxidant enzymes such as catalase. This study suggests that SSM may synergistically enhance the systemic antioxidant status of beef cattle through dual mechanisms: optimising nitrogen metabolism and regulating energy metabolism. This is achieved by modulating ruminal microbial community structure, particularly by promoting the enrichment of *g_CAG-352* and the Verrucomicrobia phylum. This discovery provides potential microbial-host interaction mechanisms supporting the application of sesame meal as a functional feed ingredient. While these correlations provide plausible microbial-related insights into the physiological effects of SSM, future studies are needed to establish causal links and clarify the functional roles of these microbial groups.

### 4.4. Economic and Environmental Implications

In addition to its nutritional advantages, the use of sesame meal in beef cattle diets can also provide economic benefits by effectively reducing feed costs. As detailed in Appendix A.1, the SSM group exhibited a lower total feed cost per head (3851.17 vs. 3958.29 CNY), primarily due to the lower market price of sesame meal relative to soybean meal. More importantly, the superior weight gain performance in the SSM group translated into significantly higher farm profit—1177.29 CNY per head compared to 660.23 CNY in the SBM group. This represents a 78.3% increase in profitability, underscoring the direct economic value of dietary sesame meal inclusion. However, the practical application of this strategy on a large scale requires consideration of market dynamics. Sesame meal, as a by-product of the grain and oil industry, has a supply chain that is less established than that of soybean meal. Its availability and price are subject to fluctuations influenced by the primary production of sesame oil for human consumption. Therefore, while our data confirms a clear cost–benefit advantage under current market conditions, we recommend that producers conduct a localized cost analysis prior to implementation. The significant reduction in the unit cost of weight gain (21.22 vs. 23.39 CNY/kg) provides a considerable buffer against potential price volatility of sesame meal. This strategy not only enhances farm-level economics but also contributes to sustainable animal production by valorizing a local agro-industrial by-product.

In terms of the environment, the addition of sesame meal to the diet significantly reduced urinary nitrogen excretion, which has profound implications for reducing greenhouse gas emissions. Although the Intergovernmental Panel on Climate Change (IPCC) provides emission factors for total fecal nitrogen, it is well known that urinary nitrogen (primarily in the form of urea) rapidly hydrolyzes to ammonia and is a more volatile and direct precursor to nitrous oxide (N_2_O) emissions compared to fecal nitrogen [47]. Our data indicate that during the experimental period, the addition of sesame meal to the diet reduced urinary nitrogen excretion by approximately 13.95 g per day per animal (or by 18%). To quantitatively assess the environmental impact, we made the following conservative estimate: the potential reduction in direct N_2_O emissions was calculated based on the total nitrogen retained in the urine. Using the IPCC default emission factor for direct N_2_O emissions from manure management (0.01 kg N_2_O-N per kg of nitrogen excreted) [71], this equates to a reduction of approximately 1.26 kg of urinary nitrogen per animal over a standard 90-day fattening period. According to the IPCC default emission factor for direct N_2_O emissions from manure management (0.01 kg N_2_O-N per kg of nitrogen excreted), this translates to an avoidance of approximately 0.0198 kg of N_2_O emissions per animal. If scaled up to a 10,000-head feedlot, this single dietary intervention could reduce direct N_2_O emissions by approximately 198 kg per production cycle. Given that the global warming potential of N_2_O is 273 times that of CO_2_, this is equivalent to a reduction of over 50 tons of CO_2_ equivalent [72,73,74,75].

### 4.5. Limitations and Future Perspectives

While this study provides evidence supporting the benefits of dietary sesame meal (SSM) in beef cattle, several limitations should be acknowledged. Firstly, the sample size employed, while sufficient to detect significant differences in primary outcomes, was relatively small. Future studies with larger cohorts are warranted to explore the sources of variation more comprehensively. Secondly, the trial was conducted during the late fattening; the long-term implications of SSM supplementation on health, reproductive performance, or multi-production cycle sustainability in beef cattle remain unknown and merit further investigation. From a mechanistic standpoint, we recognize that the improved antioxidant status was inferred from serum biochemistry. The absence of direct measurements of key bioactive compounds, such as specific lignan metabolites (e.g., sesaminol aglycones) in circulation or tissues, and the hepatic synthesis rate of glutathione, limits our ability to fully delineate the underlying metabolic pathways. Future research incorporating targeted metabolomics and isotopic tracer techniques would be valuable to directly quantify the bioavailability and metabolism of sesame-derived lignans. Finally, the practical application of SSM on a larger scale may face challenges related to the stability and sustainability of its supply chain. Variations in processing methods and seasonal availability could influence the consistent nutritional composition of SSM. Therefore, establishing standardized processing protocols and stable supply chains is crucial for the reliable utilization of SSM as a functional feed ingredient in the ruminant industry.

## 5. Conclusions

This study examined a diet where sesame meal totally replaced soybean meal, focusing on its impact on the late-phase production performance and slaughter characteristics of Angus bulls during finishing, by measuring nitrogen metabolism, antioxidant indicators, and rumen microbiota. Results indicated that antioxidant capacity was significantly enhanced by elevating the activity of antioxidant enzymes such as CAT and GSH-Px. The rumen microbial community structure was optimised through increased abundance of beneficial bacterial groups, including Verrucomicrobia, *g_CAG_352*, and Spirochaetota, with improved stability and complexity. Nitrogen metabolism efficiency improved through reduced urinary nitrogen and serum urea nitrogen levels, thereby contributing to environmental sustainability. These physiological improvements ultimately manifested as reduced feeding costs without adversely affecting growth and slaughter performance indicators. These findings reveal the application potential of sesame meal as a functional protein source rich in lignans from nutritional, physiological, and environmental perspectives, providing a viable feed strategy and scientific basis for low-carbon, high-efficiency ruminant farming.

## Figures and Tables

**Table 1:** Composition and Nutritional Value of Experimental Diets (DM basis, %).

Items	SBM	SSM
Ingredient composition		
Ground corn	39.46	45.53
Soybean meal	6.53	-
Sesame meal	-	6.54
DDGS ^1^	5.75	5.76
Premix ^2^	3.16	3.16
Wheat bran	6.46	-
Urea	0.32	0.63
NaHCO3	0.95	0.95
NaCl	0.63	0.63
MgO	0.32	0.32
Corn silage	26.61	26.65
Rice straw	9.82	9.84
Total	100	100
Chemical composition ^3^		
CP	12.78	12.82
EE	2.51	2.50
NDF	31.71	29.74
ADF	17.26	17.12
Ash	8.70	9.53
ME ^4^, MJ/kg DM	11.02	11.03

^1^ DDGS, distillers’ dried grains with solubles. ^2^ The premix contained the following per kg of diet: Cu ≥ 500 mg; Fe ≥ 1000 mg; Zn ≥ 1400 mg; Mn ≥ 1400 mg; Se ≥ 8 mg; I ≥ 6 mg; Co ≥ 8 mg; VA ≥ 220,000 IU; VD ≥ 60,000 IU; VE ≥ 1250 mg. SBM, the soybean meal group; SSM, the sesame meal group. ^3^ DM, dry matter; CP, crude protein; EE, ether extract; NDF, neutral detergent fiber; ADF, acid detergent fiber; ^4^ ME (metabolizable energy) was calculated based on the NASEM (2016) model [30].

**Table 2:** Amino Acid Content of Soybean Meal and Sesame Meal (CP basis, %).

Items ^1^	Soybean Meal	Sesame Meal
Asp	9.34	5.88
Thr	3.36	3.37
Ser	4.22	2.11
Glu	14.87	15.44
Pro	4.83	3.62
Gly	3.66	4.09
Ala	3.85	4.28
Cys	1.13	0.28
Val	3.77	3.69
Met	1.02	2.06
Ile	3.48	2.68
Leu	5.88	4.89
Tyr	2.55	2.39
Phe	3.89	3.43
His	1.99	1.60
Lys	5.11	1.13
Arg	5.37	5.71

^1^ Asp, Aspartic Acid; Thr, Threonine; Ser, Serine; Glu, Glutamic Acid; Pro, Proline; Gly, Glycine; Ala, Alanine; Cys, Cysteine; Val, Valine; Met, Methionine; Ile, Isoleucine; Leu, Leucine; Tyr, Tyrosine; Phe, Phenylalanine; His, Histidine; Lys, Lysine; Arg, Arginine.

**Table 3:** Comparison of Peak Areas for Key Lignin Compounds in Sesame Meal and Soybean Meal.

Items	Soybean Meal	Sesame Meal
sesamin	158,861.7691	794,308.8455
sesaminol	14,964.193	25,508,932.8
sesamolinol	6,450.2881	32,251.4405
sesaminol glucoside	12,066.825	61,011.9525
pinoresinol glucosides	307,695.7695	712,768.9685

**Table 4:** Effect of Complete Substitution of Sesame Meal with Soybean Meal on the Growth Performance of Angus Bulls.

Items	SBM	SSM	*p*-Value
IBW, kg	566.22 ± 10.54	565.22 ± 15.35	0.958
FBW, kg	741.56 ± 16.12	749.83 ± 25.56	0.779
ADG, kg	1.59 ± 0.09	1.69 ± 0.08	0.426
DMI, kg/d	13.21 ± 0.10	13.26 ± 0.005	0.676
FCR	8.34 ± 0.37	7.60 ± 0.48	0.288

SBM, the soybean meal group; SSM, the sesame meal group; IBW, Initial body weight; FBW, Final body weight; ADG, average daily gain; DMI, dry matter intake; FCR, feed conversion rate—the DMI/ADG ratio.

**Table 5:** Effect of Complete Replacement of Soybean Meal with Sesame Meal on the Apparent Digestibility of Angus Bulls.

Items	SBM	SSM	*p*-Value
DM, %	63.21 ± 2.81	68.14 ± 3.55	0.289
CP, %	62.24 ± 3.33	59.26 ± 4.37	0.590
EE, %	55.85 ± 3.69	65.28 ± 4.67	0.130
NDF, %	50.72 ± 3.43	57.34 ± 4.04	0.228
ADF, %	50.30 ± 0.03	54.08 ± 0.04	0.474

SBM, the soybean meal group; SSM, the sesame meal group; DM, dry matter; CP, crude protein; EE, ether extract; NDF, neutral detergent fiber; ADF, acid detergent fiber.

**Table 6:** Effect of Complete Replacement of Soybean Meal with Sesame Meal on Nitrogen Metabolism in Angus Bulls.

Items	SBM	SSM	*p*-Value
Nitrogen intake/(g/d)	266.20 ± 2.03	267.82 ± 0.05	0.509
Fecal nitrogen/(g/d)	101.16 ± 10.94	116.80 ± 11.86	0.351
Urinary nitrogen/(g/d)	76.96 ± 4.44	63.01 ± 2.68	0.020
Nitrogen retention/(g/d)	88.09 ± 11.49	88.01 ± 11.12	0.996
Nitrogen utilization/%	33.09 ± 0.04	32.86 ± 0.04	0.970

SBM, the soybean meal group; SSM, the sesame meal group.

**Table 7:** Effect of Complete Replacement of Soybean Meal with Sesame Meal on Serum Biochemical Parameters in Angus Bulls.

Items	SBM	SSM	*p*-Value
ALT, U/L	23.41 ± 1.34	27.94 ± 1.82	0.065
AST, U/L	65.26 ± 2.32	70.80 ± 3.58	0.226
ALP, U/L	124.60 ± 9.88	127.80 ± 10.54	0.827
TP, g/L	61.14 ± 3.14	61.96 ± 1.97	0.829
CHO, mmol/L	3.31 ± 0.25	3.20 ± 0.24	0.763
HDL-C, mmol/L	1.22 ± 0.06	1.16 ± 0.10	0.608
LDL-C, mmol/L	0.73 ± 0.06	0.68 ± 0.04	0.497
NEFA, mmol/L	0.21 ± 0.03	0.21 ± 0.01	0.813
BHBA, mmol/L	0.21 ± 0.02	0.21 ± 0.02	0.880
UREA, mmol/L	4.03 ± 0.18	3.44 ± 0.10	0.016
CREA, mmol/L	106.26 ± 5.66	115.86 ± 3.42	0.166
GLU, mmol/L	4.21 ± 0.26	4.61 ± 0.32	0.345
TG, mmol/L	0.13 ± 0.02	0.11 ± 0.01	0.357
ALB, g/L	32.43 ± 0.85	34.34 ± 0.52	0.077
GLB, g/L	28.72 ± 2.56	28.33 ± 1.41	0.898
ALB / TP	0.55 ± 0.01	0.54 ± 0.01	0.607
GLB / TP	0.45 ± 0.01	0.46 ± 0.01	0.607
ALB / GLB	1.23 ± 0.03	1.20 ± 0.05	0.689

SBM, the soybean meal group; SSM, the sesame meal group; TP, total protein; CHO, total cholesterol; AST, aspartate aminotransferase; TG, triglycerides; HDL−C, high−density lipoprotein cholesterol; LDL−C, low−density lipoprotein cholesterol; UREA, urea nitrogen; NEFA, non−esterified fatty acids; GLU, glucose; BHBA, β-hydroxybutyric acid; CREA, creatinine; ALP, alkaline phosphatase; ALT, alanine aminotransferase; ALB, albumin; GLB, globulins, GLB = TP−ALB.

**Table 8:** Effect of Complete Replacement of Soybean Meal with Sesame Meal on Serum Antioxidant Capacity in Angus Bulls.

Items	SBM	SSM	*p*-Value
CAT, U/mL	0.50 ± 0.11	0.91 ± 0.14	0.043
SOD, U/mL	35.06 ± 1.99	32.41 ± 2.21	0.389
T-AOC, Trolox mM	0.79 ± 0.05	0.78 ± 0.01	0.900
MDA, nmol/mL	2.52 ± 0.11	2.40 ± 0.09	0.421
GSH-Px, ng/mL	219.76 ± 12.42	252.12 ± 11.66	0.076
ROS, IU/mL	18.58 ± 2.73	19.55 ± 3.95	0.839
OSI	24.87 ± 4.31	24.50 ± 5.24	0.957

SBM, the soybean meal group; SSM, the sesame meal group; CAT, catalase; SOD, superoxide dismutase; MDA, malondialdehyde; ROS, reactive oxygen species; GSH−Px, glutathione peroxidase; T-AOC, total antioxidant capacity; OSI, oxidative stress index, OSI= ROS/T−AOC.

**Table 9:** Effect of Complete Replacement of Soybean Meal with Sesame Meal on Ruminal Fermentation in Angus Bulls.

Items	SBM	SSM	*p*-Value
pH	6.31 ± 0.11	6.58 ± 0.13	0.131
Acetate, mmol/L	88.30 ± 6.09	78.60 ± 6.13	0.278
Propionate, mmol/L	21.32 ± 1.96	17.19 ± 1.66	0.128
Isobutyrate, mmol/L	0.87 ± 0.09	0.78 ± 0.04	0.372
Butyrate, mmol/L	13.88 ± 1.32	13.18 ± 1.36	0.714
Isovalerate, mmol/L	1.99 ± 0.22	1.91 ± 0.20	0.797
Valerate, mmol/L	0.42 ± 0.04	0.38 ± 0.03	0.353
TVFA, mmol/L	126.79 ± 9.22	112.05 ± 8.97	0.269
A:P ratio	4.29 ± 0.24	4.69 ± 0.22	0.242

SBM, the soybean meal group; SSM, the sesame meal group; TVFA, total volatile fatty acids; A:P ratio, Acetate: Propionate ratio.

**Table 10:** Effect of Complete Replacement of Soybean Meal with Sesame Meal on Slaughter Performance and Chemical Composition in Angus Bulls.

Items	SBM	SSM	*p*-Value
Slaughter Performance			
Live weight before slaughter, kg	696.00 ± 18.78	691.50 ± 26.80	0.889
Net meat weight, kg	270.25 ± 13.23	277.50 ± 8.34	0.677
Net Meat Ratio, %	38.76 ± 0.01	40.25 ± 0.01	0.420
Perirenal fat accumulation, kg	8.30 ± 1.50	9.20 ± 0.78	0.640
Perirenal fat proportion, BW%	1.17 ± 0.00	1.33 ± 0.00	0.550
Chemical Composition			
DM, %	26.20 ± 0.00	26.55 ± 0.00	0.774
CP, %DM	77.51 ± 2.81	80.32 ± 1.97	0.461
EE, %DM	13.09 ± 0.01	14.96 ± 0.02	0.426

SBM, the soybean meal group; SSM, the sesame meal group; BW, body weight.

## Data Availability

Original manuscript of this study is included in the article, and further information is available upon reasonable request to the corresponding author. The raw sequencing data supporting the findings of this study have been deposited in the NCBI Sequence Read Archive (SRA) under the BioProject accession number PRJNA1348615. The data are publicly accessible at https://www.ncbi.nlm.nih.gov/.

## Abbreviations

The following abbreviations are used in this manuscript:

ADF	Acid Detergent Fibre
Ash	Crude Ash
ADG	Average daily weight gain
AIA	Acid Insoluble Ash
ALB	Albumin
ALP	Alkaline Phosphatase
ALT	Alanine Transaminase
AST	Aspartate aminotransferase
BHBA	β-Hydroxybutyric Acid
CAT	Catalase
CHO	Total Cholesterol
CP	Crude Protein
CREA	Creatinine
DM	Dry Matter
DMI	Dry matter intake
EE	Ether Extract
FCR	Feed conversion ratio
FBW	Final body weight
GLB	Globulin
GLU	Glucose
GSH-Px	Glutathione Peroxidase
HDL-C	High-Density Lipoprotein Cholesterol
LDL-C	Low-Density Lipoprotein Cholesterol
IBW	Initial Body Weight
MDA	Malondialdehyde
ME	Metabolizable Energy
NDF	Neutral Detergent Fibre
NEFA	Non-Esterrified Fatty Acid
OSI	Oxidative stress index
PCoA	Principal Co-ordinates Analysis
ROS RDA	Reactive Oxygen Species Redundancy Analysis
SOD	Reactive Oxygen Species
SEM	Standard error of mean
T-AOC	Total Antioxidant Capacity
TG	Triglyceride
TMR	Total Mixed Ration
TP	Total Protein
VFA	Volatile Fatty Acid

## Appendix A

### Appendix A.1

**Table A1:** Effect of Complete Replacement of Soybean Meal by Sesame Meal on the Economics of Fattening Angus bulls.

Items	SBM	SSM
Feed cost ^1^, CNY ^5^/head	3958.29	3851.17
Weight gain income ^2^, CNY/head	4818.52	5069.66
Unit weight gain cost ^3^, CNY/kg	23.39	21.22
Farm pofit ^4^, CNY/head	660.23	1177.29

^1^ Feed cost = feed price × DMI × number of days in the experiment; ^2^ Weight gain income = total weight gain in the test period× price of live cattle (27 CNY/kg); ^3^ Unit weight gain cost = Feed cost/Total weight gain during the test period; ^4^ Farm profit = total weight gain in the trial period × selling price of live cattle (27 CNY/kg)-feed cost; ^5^ CNY, Chinese Yuan. SBM, the soybean meal group; SSM, the sesame meal group.

### Appendix A.2

**Table A2:** The α Diversity of Rumen Bacteria.

Items	SBM	SSM	*p*-Value
Ace	2619.66 ± 256.39	2621.05 ± 220.00	0.860
Shannon	6.85 ± 0.07	6.84 ± 0.06	0.724

SBM, the soybean meal group; SSM, the sesame meal group.

### Appendix A.3

**Table A3:** The Relative Abundance of Bacterial Communities at the Phylum Level.

Items	SBM	SSM	*p*-Value
Actinomycetota	537.22 ± 46.32	498.56 ± 29.54	0.492
Bacillota	18,567.78 ± 402.97	18,262.56 ± 529.57	0.653
Bacteroidota	17,148.56 ± 407.22	7252.89 ± 469.55	0.869
Candidatus_Eremiobacterota	4.22 ± 0.81	4.11 ± 1.12	0.937
Chloroflexota	44.56 ± 7.73	82.11 ± 14.65	0.042
Cyanobacteriota	18.11 ± 2.52	35.00 ± 5.27	0.011
Elusimicrobiota	10.89 ± 2.81	19.56 ± 3.07	0.054
Fibrobacterota	185.78 ± 12.08	163.00 ± 20.28	0.349
Patescibacteria	627.89 ± 31.79	613.33 ± 19.97	0.703
Pseudomonadota	433.56 ± 35.01	265.56 ± 9.47	<0.001
SAR324_clade (Marine_group_B)	5.11 ± 1.58	6.67 ± 0.96	0.411
Spirochaetota	474.78 ± 51.06	686.89 ± 77.37	0.036
Synergistota	4.11 ± 1.33	8.78 ± 2.19	0.087
Thermodesulfobacteriota	99.44 ± 5.71	150.89 ± 8.30	<0.001
Verrucomicrobiota	306.33 ± 28.71	400.56 ± 29.89	0.037
unclassified_k__norank_d__Bacteria	206.11 ± 14.65	216.11 ± 24.72	0.560

SBM, the soybean meal group; SSM, the sesame meal group.

### Appendix A.4

**Table A4:** The Relative Abundance of Bacterial Communities at the Genus Level.

Items	SBM	SSM	*p*-Value
*g_Rikenellaceae_RC9_gut_group*	3083.22 ± 64.06	3443.00 ± 205.86	0.115
*g_Ruminococcus*	3470 ± 48.91	3577.33 ± 118.07	0.416
*g_Xylanibacter*	6496.33 ± 497.90	6592.33 ± 651.67	0.908
*g_norank_f_F082*	2114.89 ± 107.77	2046.33 ± 99.25	0.646
*norank_f_Lachnospiraceae*	128.22 ± 7.60	173.78 ± 8.68	0.001
*g__norank_o__RF39*	615.44 ± 55.69	693.78 ± 46.16	0.295
*g__Christensenellaceae_R-7_group*	1341.89 ± 35.48	1056.78 ± 71.04	0.002
*g__norank_o__Clostridia_UCG-014*	758.89 ± 61.31	712.56 ± 47.16	0.558
*g__Lachnospiraceae_NK3A20_group*	1158.67 ± 42.33	1081.56 ± 57.31	0.295
*g__unclassified_f__Lachnospiraceae*	648.89 ± 30.98	600.33 ± 23.16	0.227
*g__norank_f__[Eubacterium]_coprostanoligenes_group*	722.89 ± 26.31	676.44 ± 25.99	0.227
*g__unclassified_c__Clostridia*	1135.56 ± 76.17	1083.22 ± 59.45	0.596
*g__norank_f__Bacteroidales_RF16_group*	1209.11 ± 46.32	1439.33 ± 98.83	0.051
*g__norank_f__Muribaculaceae*	1293.89 ± 185.48	710.44 ± 55.98	0.008
*g__Prevotellaceae_UCG-003*	518.44 ± 48.25	643.44 ± 58.56	0.119
*g__Treponema*	462.56 ± 51.57	663.33 ± 75.38	0.043

SBM, the soybean meal group; SSM, the sesame meal group.
